# The Role of Synthetic Meshes in Revision Surgery After Breast Augmentation: A Personal Experience

**DOI:** 10.3390/jcm14175978

**Published:** 2025-08-24

**Authors:** Isabel Zucal, Ester Cresta, Laura De Pellegrin, Andrea Weinzierl, Yves Harder

**Affiliations:** 1 Department of Plastic, Reconstructive and Aesthetic Surgery and Hand Surgery, University Hospital of Lausanne (CHUV), 1005 Lausanne, Switzerland; 2 Department of Plastic, Reconstructive and Aesthetic Surgery, Ente Ospedaliero Cantonale (EOC), 6900 Lugano, Switzerland; 3 Department of Plastic and Hand Surgery, Inselspital Bern, 3010 Bern, Switzerland; 4 Department of Plastic and Hand Surgery, Universitätsspital Zürich (USZ), 8091 Zürich, Switzerland; 5 Faculty of Biology and Medicine, University of Lausanne (UNIL), 1015 Lausanne, Switzerland

**Keywords:** breast, breast augmentation, mammoplasty, implants, surgical mesh, surgical revision

## Abstract

**Background/Objectives**: The breast implant exchange/explantation rate has been increasing in recent years due to various types of long-term complications or adverse effects, such as implant migration, rippling, or capsular contracture. To reduce complications such as migration and/or implant–pocket mismatch, surgical meshes may provide implant support. Here, we present a case series about the use of a non-absorbable synthetic bra-shaped mesh in revision surgery of the breast, using implants that do not adhere to the surrounding tissues. **Methods**: In this retrospective case series, eight patients underwent breast revision surgery between 2021 and 2024 due to implant-related long-term complications following aesthetic surgery. Surgical revision included implant exchange, total or partial capsulectomy, creation of a pre-pectoral implant pocket for the new implant, and positioning of the non-absorbable synthetic mesh, acting as an internal support for the implants. BREAST-Qs were collected from all patients. **Results**: Of the eight patients included, the following symptoms were observed: symptomatic capsular contracture (*n* = 3), implant migration (*n* = 4), and breast animation deformity (*n* = 2). After revision surgery, during the follow-up period of 6–42 months, neither infection nor seroma occurred. No implant-related complications were registered. The BREAST-Q analysis revealed the highest patient satisfaction in the domain “satisfaction with the implants” (median score 87.5%). **Conclusions**: In revision surgery after breast augmentation, the synthetic, non-absorbable and titanized pocket-like mesh may provide implant support and avoid recurrence of complications related to implant position. However, due to the small and heterogeneous patient group, larger studies are needed to validate these preliminary findings.

## 1. Introduction

Implant-based breast augmentation is one of the most common aesthetic surgical procedures worldwide [1]. Nevertheless, many patients experience a decay of the initial surgical outcome over time due to long-term complications, usually related to the implant. For this reason, revision rates after breast augmentation are high, reaching around 36% [2]. According to recent statistics from the International Society of Aesthetic Plastic Surgery (ISAPS) from 2023, the percentage of patients undergoing implant removal due to various factors is approximately 18% [3]. The use of synthetic meshes is a promising option to address complications, such as mismatch between the breast pocket and the implant’s footprint, implant migration, and thereby aesthetic dissatisfaction. Furthermore, these meshes seem to be particularly interesting to enhance the stability of smooth implants that do not adhere to the adjacent tissues. This is of importance, since the use of smooth implants has progressively increased because of the well-documented association between macrotextured implants and the development of implant-associated anaplastic large cell lymphoma (BIA-ALCL) [4].

Revisional breast surgery following malposition of implants represents a significant surgical challenge, particularly in preventing recurrence of the malposition. Once the implant pocket has been distorted or over-dissected, achieving long-term stabilization of the implant within thedesired anatomical position becomes increasingly complex. This is especially true in cases where the soft tissue support is insufficient or compromised due to prior surgeries. In recent years, the pre-pectoral placement of implants, fully enveloped by either a biological or a synthetic mesh have been described [5]. Most reports and studies regarding the application of surgical meshes in breast surgery focus on reconstructive settings, particularly in cases of implant–pocket mismatch following mastectomy, whereas in the context of aesthetic revisional breast surgery, there is a lack of literature.

Currently, meshes utilized in breast surgery are classified as either biologically derived or synthetically manufactured [6]. Biological meshes originate from decellularized human or animal tissues and serve as a collagen-rich scaffold that facilitates tissue regeneration through host integration, remodeling, and de novo collagen synthesis [7]. In contrast, synthetic meshes are composed of polymeric materials and are categorized based on their degradation profiles into absorbable and non-absorbable types, such as titanium-coated polypropylene meshes [6]. In this regard, the TiLOOP Bra Pocket^®^ is a non-absorbable polypropylene mesh with a titanized surface, which is tolerated well by the body and minimizes inflammatory reactions [8]. Synthetic meshes, according to the current literature, are at least equivalent to biological meshes in several clinical outcomes, such as stable implant position, which provides a compelling argument for prioritizing the use of synthetic meshes in implant-based breast surgery [9].

The aim of this case series was to describe a single-center experience of the use of surgical meshes in breast revision surgery using smooth implants, in order to stabilize them and to possibly avoid recurrence of implant displacement. Moreover, patient satisfaction was evaluated.

## 2. Materials and Methods

Eight consecutive patients undergoing revision surgery after breast augmentation were included and retrospectively analyzed. Thereby, implant exchange was undertaken, changing the pocket from sub- to pre-pectoral if needed and supported with a surgical mesh (TiLOOP^®^ Bra Pocket, pfm medical gmbh, Köln, Germany). Due to the retrospective study design without matched controls and small sample size, the findings are descriptive and cannot be generalized or used to infer causality. Seven patients underwent surgery at the Regional Hospital of Lugano, Switzerland, and one was operated on in Düsseldorf, Germany. All patients were operated on by a single senior surgeon between November 2021 and April 2024 and gave written consent to the evaluation and publication of their data and photographic material. Patient inclusion was performed according to the Declaration of Helsinki and the Local Ethics Committee gave the consent to the publication of this case series. Since the product used is certified by the European Medicine Agency (EMA) and therefore on the European market, the case series was not seen as a medical product study. Also, it has already been used for a few years in breast reconstruction surgery. Consequently, the Local Ethics Committee classified it as a quality control (since the BREAST-Qs were also handed out to assess patient satisfaction) and approved the publication of this case series, according to the patient consents.

The surgical revision included implant removal and partial or complete capsulectomy where needed, refixation of the major pectoral muscle if the implant was partially or totally sub-pectoral, creation of a new pre-pectoral breast pocket if required, insertion of the TiLOOP^®^ Bra Pocket after pocket irrigation (3 × povidone-iodine (Betadine^®^), 3 × NaCl 0.9%), fixation of the mesh with absorbable sutures, and insertion of the new implants. Surgical drains were placed routinely and were removed as soon as the daily drainage volume was below 30–40 mL. Surgical wounds were closed using absorbable sutures. A compressive bra was used for 24 h daily for six weeks post-operatively. Regular follow-up visits were scheduled after 2 weeks, 1 month, 3 months, 6 months, and 12 months after surgery, and thereafter on a yearly basis.

The post-operative course of the patients was recorded at the follow-up consultations (standardized photographs to assess the breast’s morphology over time), and BREAST-Q questionnaires (Version 2.0 ©, Augmentation Module) [10] were collected from all patients at least 6 months after surgery to report patient-reported outcome measures (PROMs). The domains of the BREAST-Q included psychosocial well-being, sexual well-being, satisfaction with the breasts, physical well-being of the chest, satisfaction with the implants, satisfaction with the outcome, satisfaction with the information, satisfaction with the surgeon, satisfaction with the medical staff, and satisfaction with the office staff.

## 3. Results

All patients had previously undergone breast augmentation elsewhere because of breast hypotrophy, and three of them also suffered from contemporary breast ptosis requiring additional mastopexy. Moreover, one patient suffered from an asymmetric funnel chest deformity (“pectus excavatum”), requiring the additional positioning of a custom-made sternal implant.

After a median of 3 years (range: 1–16 years), patients underwent surgical revision due to the following indications: three patients presented with symptomatic capsular contracture (Baker grade III or IV), four presented implant migration (two with bottoming out of the implants = caudal implant migration, one with lateral implant migration, and one with post-surgical synmastia = medial migration), and one with symptomatic breast animation deformity. The explanted implants included Motiva implants in five cases (nanotextured), as well as McGhan (macrotextured), Polytech (microtextured), and Silimed (microtextured) in one case each. They were replaced with nanotextured ergonomic implants (Ergonomix^®^; Establishment Labs, Costa Rica) in seven patients and microtextured, lightweight implants (B-Lite, Polytech, Germany) in one patient. A plane change from sub-pectoral to pre-pectoral implant positioning was performed in five cases. Concomitant procedures included mastopexy in five cases (3x peri-areolar, 2x inverted T-mastopexy), as well as reconstruction of the intermammary fold (“cleavage area”), reconstruction of the inframammary fold, and bilateral autologous fat grafting in one case, respectively. The post-operative follow-up period ranged from 6 to 42 months. Non-implant-related complications included a thrombophlebitis at the inframammary fold in one patient and an episode of chest pain in another patient (most likely of musculoskeletal origin). Implant-related complications such as breast animation deformity, severe capsular contracture (Baker grade III or IV), or implant displacement were not observed during the follow-up period. However, mild capsular contracture (grade II) was observed in three cases after a follow-up of 36, 18, and 8 months, respectively. The inserted mesh was not palpable during the follow-up as reported by patients and the surgeon.

All patients completed the post-operative BREAST-Q: The domains with the highest satisfaction scores were satisfaction with the implants (median score 87.5%), the surgeon, the medical staff, and the office staff (all median score 100%). A low rate of breast and chest pain (median score 5–7%) was reported by all patients. The lowest satisfaction was reported with regard to sexual well-being (median score 45%).

### Case Presentation

A detailed case description is provided for cases 1, 3, and 7. An overview of all cases (1–8) is given in Table 1.

Case 1

**Figure 1:**
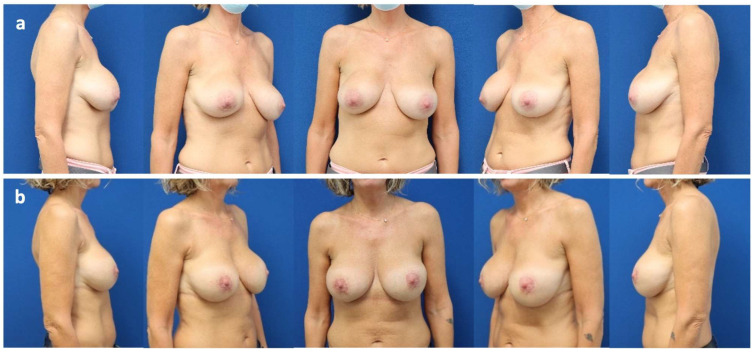
Case 1. (**a**) 45-year-old patient with bilateral symptomatic capsular contracture (Baker grade III) and breast ptosis pre-operatively (Regnault grade I). (**b**) Post-operative status at 36 months after revisional breast surgery including pre-pectoral implant exchange (Motiva Ergonomix^®^ ERSF 315Q), mesh positioning (TiLOOP Pocket Bra^®^), autologous fat grafting, and peri-areolar mastopexy. The long-term follow-up is shown in the Appendix A, Figure A1.

**Figure A1:**
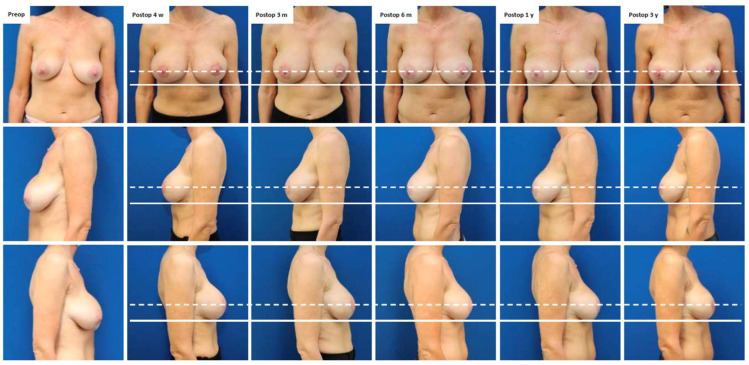
Case 1. Evolution over time with a stable nipple and inferior pole position over 3 years of follow-up. The dashed and solid line show stability of nipple position respectively inferior pole over time.

45-year-old patient who underwent pre-pectoral breast augmentation due to bilateral breast hypotrophy in 2015. Over time, the patient developed symptomatic capsular contracture (Baker grade III) and bilateral breast ptosis (Regnault grade I) that resulted in revision surgery in 2021; the implants were exchanged and positioned in a pre-pectoral plane, supported by a bra-like shaped mesh. Moreover, bilateral autologous fat grafting and peri-areolar mastopexy were associated. After a follow-up of 36 months, the patient presents mild capsular contracture (Baker grade II) bilaterally, without any other implant-related problems (Figure 1, Figure A1).

Case 3

**Figure 2:**
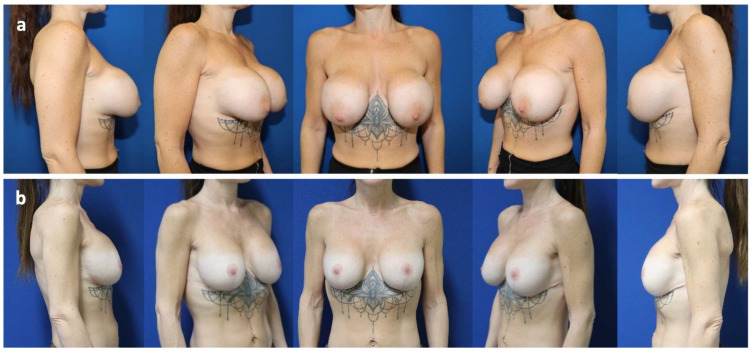
Case 3. (**a**) 24-year-old patient presenting with bilateral symptomatic capsular contracture (Baker grade IV), associated with waterfall deformity. (**b**) Post-operative status at 18 months after revision surgery, including change of plane from sub- to pre-pectoral, implant exchange (Motiva Ergonomix^®^ E2SM 375Q), mesh positioning (TiLOOP Pocket Bra^®^) and inverted T-mastopexy.

24-year-old patient who underwent pre-pectoral breast augmentation due to bilateral breast hypotrophy in 2010. She presented with bilateral symptomatic capsular contracture (Baker grade IV) associated with waterfall deformity that led to revision surgery consisting of bilateral implant exchange associated with an inverted T-mastopexy and insertion of the bra-like shaped mesh in 2023. After a follow-up of 18 months, an asymptomatic mild capsular contracture (Baker grade II) was observed, but no implant migration (Figure 2).

Case 7

**Figure 3:**
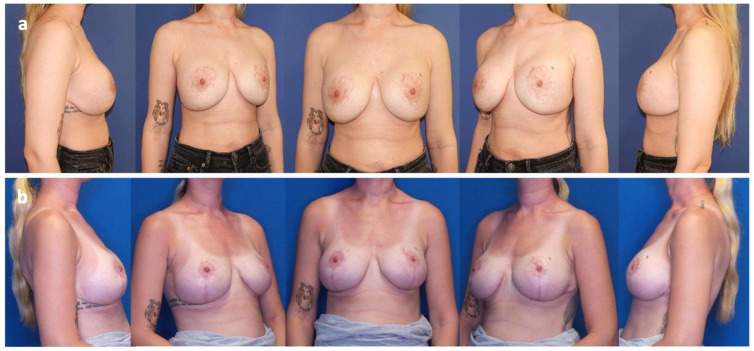
Case 7. (**a**) 24-year-old patient presenting with bottoming out and lateralization of the left implant. (**b**) Post-operative status at 12 months after revision surgery, including pre-pectoral implant exchange (Motiva Ergonomix^®^ E2SM 300Q), capsulotomies, and mesh positioning (TiLOOP Pocket Bra^®^).

24-year-old patient, who underwent mastopexy in 2021 for bilateral breast hypotrophy and ptosis, followed by sub-pectoral breast augmentation one year later. Subsequently, she reported implant malposition (latero-caudal migration/bottoming out), breast animation deformity, and asymmetry. In 2024, bilateral implant exchange with a change of plane from sub-pectoral to pre-pectoral was performed with the insertion of the bra-shaped mesh, as well as an inverted T-mastopexy. At the 1-year follow-up, no recurrence of ptosis was observed (Figure 3).

## 4. Discussion

The results shown herein demonstrate that synthetic meshes may be a helping tool in secondary aesthetic breast surgery to retain the implant in its ideal position after revising its pocket, especially with the use of smooth implants. They reduce the rate of potential complications related to the implant position, as well as their recurrence.

Post-surgical bottoming out, lateral or medial implant migration, breast animation deformity, as well as symptomatic capsular contracture have been the complications requiring surgical revision in this series. Capsular contracture, which may also result in cranial implant displacement [11], represented one of the most frequently observed implant-related complications in this series, which is consistent with current data resulting from aesthetic breast surgery [12]. Additionally, breast animation deformity, commonly associated with sub-pectoral implant placement, can contribute to progressive implant malposition over time due to repetitive contraction of the pectoral muscles [2,13], particularly if the implants have a smooth or microtextured surface that does not adhere to the adjacent tissues above and under the implant. Since currently there is a trend to move from macrotextured towards microtextured or smooth implants due to the rare risk of BIA-ALCL, implant stabilization becomes a growing issue [14]. Ideally, breast anatomy must be preserved during the initial surgery, especially in aesthetic breast surgery, including the supportive Cooper’s ligaments between the skin and the glandular tissue all around the breast. Though, surgery as well as medical conditions following massive weight loss may harm or weaken these tissues, resulting in a compromised future pocket for the implant. The application of a supportive mesh has been shown to reduce the risk of implant rotation or displacement by providing structural reinforcement [15]. The current results show that the surgical approach that combines smooth or microtextured implants with a synthetic, non-absorbable bra-shaped mesh allows for a correct and stable implant positioning after pre-pectoral neo-pocket dissection. Indeed, advantages of the pre-pectoral over the sub-pectoral implant pocket are described well in the literature: For example, Salgarello et al. report that converting from a sub-pectoral to a pre-pectoral plane is a beneficial technical choice, likewise after mastectomy, as it is associated with a lower rate of post-operative pain, the absence of breast animation deformity, and a reduced rate of implant displacement [16]. However, pre-pectoral implant placement has a certain risk of increased implant visibility [2]. In fact, the implant showing rippling raises the potential rate of revision surgeries [17].

The current experience is in line with other studies, confirming that the use of a mesh may improve long-term implant stability and eventually reduce the need for re-operation for implant displacement. Tellarini et al. show that the risk of implant rotation and dislocation is reduced by half when using the synthetic, non-absorbable bra-shaped mesh as a lower-pole support when compared to unsupported implants [18]. Further, this type of mesh is as efficient to support implants when compared to biological meshes or acellular dermal matrices (ADMs) [17]. In fact, Quah et al. could demonstrate that biological meshes carry a higher risk of seroma, infection, wound breakdown, and implant rotation when compared to synthetic meshes [19]. This is consistent with the present case series that showed neither significant inflammatory reaction nor persistent seromas or infection. Furthermore, the results presented herein confirm that the synthetic, non-absorbable bra-shaped mesh may be used in different clinical conditions without inducing higher grades of symptomatic capsular contracture (Baker III and IV). Three of eight patients developed a non-symptomatic Baker grade II capsular contracture. In this regard, Casella et al. could only show a 4% rate of grade III and IV capsular contracture in implant-based breast reconstruction with mesh support after mastectomy [20].

The post-operative BREAST-Q questionnaire revealed a high level of satisfaction with the implants and the quality of medical care received. The resolution of previous complications and complaints and the improvement of the aesthetic outcome likely contributed to these high scores. Of interest, the lowest score was recorded in the domain of sexual well-being, in line with other studies focused on the use of this synthetic, non-absorbable bra-shaped mesh supporting implants in implant-based breast reconstruction [21]. This rather surprising result—despite the significant functional and aesthetic improvement of the breasts—may be attributed to prior unpleasant surgical experiences that influenced the patient’s body image and sensitivity to aesthetic outcomes.

Although this case series provides a low level of evidence, we believe that it may serve as a future basis for larger clinical studies to explore this specific topic further. Nevertheless, all patients have been operated on by the same surgeon, and therefore, operator-related biases may be excluded. However, the average follow-up period was approximately 19 months, with quite some variability, which makes it challenging to draw definitive conclusions regarding the true duration of the observed benefits of the mesh support and the potential occurrence of long-term complications. Furthermore, it must be noted that revisional surgery due to implant-associated complications after breast augmentation increases with time. In this regard, one group will opt for an explantation of the implants to get rid of the foreign body, whereas the other group—still the majority—will go for implant exchange, considering that two current trends are observed: 1. change of the plane from sub- to pre-pectoral and 2. the use of smooth or microtextured implants in the majority of the cases, eventually at risk of migration or dislocation. In this regard, polyurethane implants have been shown to be a valid alternative to implants supported by meshes, preventing recurrence of capsular contracture and implant displacement by bio-integrating with the surrounding tissues, as described by Hamdi et al. [22,23].

Despite the encouraging results and low complication rates in the present case series, the use of synthetic, non-absorbable bra-shaped meshes is not completely free from complications. Paepke et al. studied 231 patients who underwent breast reconstruction with this synthetic, titanized, non-absorbable bra-shaped mesh and observed the following complication rates: superficial hematoma (9.5%), need for re-interventions with mesh removal (7.8%), skin infection (6.1%), seroma (4.8%), skin necrosis (3.9%), partial nipple necrosis (3.5%), and capsular fibrosis (2.2%) [24]. In this regard, it must be emphasized that mastectomy itself carries a certain risk of complications that are neither related to the implant, nor to the mesh, which may not correspond to the same risk profile seen in the exchange of implants for aesthetic purposes, since in these cases the tissues are almost never exposed to ischemic risks, as may be the case after mastectomy. This may explain the rather high rate of hematoma, infection, and tissue necrosis that are device (implant and mesh)-independent [24]. A subsequent study conducted by Dieterich et al. suggested advantages in the use of non-absorbable bra-shaped meshes compared to unsupported implants in implant-based breast reconstruction, reporting an overall complication rate of 14.6% with the titanized, synthetic, non-absorbable bra-shaped mesh versus 28.6% in the control group without mesh [21]. Especially the rate of capsular contracture in the group of the synthetic non-absorbable bra-shaped mesh was approximately 3.8 times lower compared to the group without support (4.4% vs. 16.7%) [21]. Finally, in 2024, Guo et al. conducted a meta-analysis (7 studies including 1203 cases), proving a significant reduction in the risk of complications with the use of the non-absorbable bra-shaped meshes compared to other meshes, observing a significant reduction of infection, seroma, red breast syndrome and capsular contracture [25].

At this point it must be underlined that the rather young age of treated women leads to a higher risk of developing breast cancer and therefore being exposed to radiotherapy, especially after lumpectomy in the context of breast conserving therapy. In this case, the non-absorbable synthetic mesh would get irradiated, as would the implant. Although Quah et al. reported that adjuvant radiotherapy would not significantly impact the incidence of short-term postoperative complications, their study did not assess long-term outcomes such as capsular contracture [19]. In contrast, Casella et al. observed a capsular significant capsular contracture (Baker grade III and IV) in 4% of the patients undergoing implant-based breast reconstruction with implants and non-absorbable surgical meshes, of which all patients reporting grade IV capsular contracture had undergone radiotherapy [20]. More recently, Ohlinger et al. described a capsular fibrosis rate of 3.6% using non-absorbable, surgical meshes in immediate, sub-pectoral breast reconstruction and radiotherapy, which was lower compared to the 7.7% rate described with the use of ADM [26].

### Limitations of the Study

Due to the study design lacking a control group, the small sample size and patient heterogeneity regarding initial diagnosis, the herein demonstrated findings are only descriptive and cannot be generalized. Moreover, concomitant procedures such as mastopexy may influence the outcome.

Although some reviews found no striking evidence of the advantages of the use of surgical meshes in breast surgery in general [27,28], studies are very heterogeneous (different surgical techniques: reconstruction, mastopexy, breast reduction–mastopexy–augmentation; different surgical meshes: ADM, biological, synthetic meshes; different previous operations) and therefore the few, current findings cannot be generalized. Larger studies for each type of patient cohort (reconstruction vs. reduction vs. augmentation vs. revision) are demanded to draw objective conclusions.

## 5. Conclusions

In this small cohort, the use of the mesh was associated with satisfactory patient-reported outcomes and no recurrence of major smooth implant malposition during the follow-up period. However, these preliminary findings require validation in larger, controlled trials to determine the efficacy and safety of this technique.

## Figures and Tables

**Table 1:** Case presentations 1–8.

Case No.	Age (Years)	Previous Breast Surgery, Year	Symptoms Leading to Surgical Revision	Revision Surgery, Year	Follow-Up (Months)	Implant-Related Complications After Revision
1	45	Pre-pectoral breast augmentation (2015)	Symptomatic capsular contracture (Baker grade III) and bilateral breast ptosis (Regnault grade I)	Bilateral implant exchange, mesh insertion and peri-areolar mastopexy (2021) (Figure 1, Figure A1)	36	Mild capsular contracture (Baker grade II)
2	40	Peri-areolar mastopexy and sub-pectoral breast augmentation (dual plane II: 2021)	Severe breast pain	Bilateral implant exchange, change of plane from partially sub-pectoral to pre-pectoral, mesh insertion and peri-areolar mastopexy (2022) (Figure A2)	42	No, pain resolved
3	24	Pre-pectoral breast augmentation (2010)	Symptomatic capsular contracture (Baker grade IV) and waterfall deformity	Bilateral implant exchange associated with inverted T-mastopexy and mesh insertion (2023)	18	Mild capsular contracture (Baker grade II)
4	43	Sub-pectoral breast augmentation (dual-plane II: 1999), capsulectomy and breast implant exchange (2010)	Inferior implant displacement (bottoming out) and lateralization of left implant	Bilateral implant exchange, change of plane from partially sub-pectoral to pre-pectoral and mesh insertion (2023)	18	No
5	37	Sub-pectoral breast augmentation (dual-plane II: 2022)	Inferior implant displacement (bottoming out)	Bilateral implant exchange, change of plane from partially sub-pectoral to pre-pectoral, reconstruction of inframammary fold and mesh insertion (2024)	6	No
6	30	Pre-pectoral breast augmentation and autologous fat grafting (2022) for breast asymmetry after pectus excavatum correction	Synmastia (medial implant displacement)	Reconstruction of intermammary fold, bilateral exchange of breast implants and mesh insertion (2024)	11	No
7	24	Mastopexy (2021) and sub-pectoral breast augmentation (dual-plane II: 2022)	Implant malposition (latero-caudal displacement/bottoming out), breast animation deformity, and asymmetry	Bilateral implant exchange, change of plane from partially sub-pectoral to pre-pectoral, mesh insertion and inverted T mastopexy	12	No
8	37	Peri-areolar mastopexy and sub-pectoral breast augmentation (2008)	Symptomatic capsular contracture (Baker grade IV), waterfall deformity and bilateral implant rupture	Bilateral implant exchange with change of plane from partially sub-pectoral to pre-pectoral, mesh insertion and peri-areolar mastopexy (2024)	8	Mild capsular contracture (Baker grade II)

**Figure A2:**
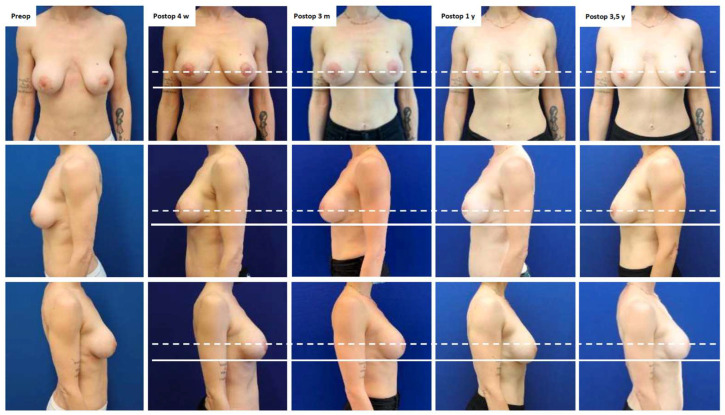
Case 2. Evolution over time with a stable nipple and inferior pole position over 3.5 years of follow-up. The dashed and solid line show stability of nipple position respectively inferior pole over time.

## Data Availability

The data presented in this study are available on request from the corresponding author due to privacy reasons.
